# Role of Embinin in the reabsorption of nucleus pulposus in lumbar disc herniation: Promotion of nucleus pulposus neovascularization and apoptosis of nucleus pulposus cells

**DOI:** 10.1515/biol-2022-0878

**Published:** 2024-05-29

**Authors:** Yingying Meng, Wei Liu, Haifeng Liu, Chengwei Yu

**Affiliations:** Department of Acupuncture and Moxibustion Massage, Wenzhou TCM Hospital of Zhejiang Chinese Medical University, Wenzhou, Zhejiang Province, 325000, China; Department of Rehabilitation, Wenzhou TCM Hospital of Zhejiang Chinese Medical University, Wenzhou, Zhejiang Province, 325000, China; Department of Orthopedics, Wenzhou TCM Hospital of Zhejiang Chinese Medical University, No. 9 Jiaowei Road, Lucheng District, Wenzhou, Zhejiang Province, 325000, China

**Keywords:** lumbar disc herniation, Embinin, neovascularization, cAMP pathway, apoptosis

## Abstract

Reabsorption of the nucleus pulposus (NP) in lumbar disc herniation (LDH) refers to the natural absorption or even complete disappearance of LDH. In order to better treat LDH, it is necessary to further study its mechanism and develop new therapeutic drugs. Clematidis Radix Et Rhizoma is a ranunculus family plant which has multiple biological activities, and Embinin is one of its bioactive ingredients. However, its effects on LDH were unclear. In this study, the role of Embinin was investigated in LDH rat models. LDH model was established by lumbar epidural insertion of tail disc. Our results showed that Embinin promoted lumbar disc neovascularization, induced apoptosis of NP cells in LDH rats, and promoted lumbar disc resorption. Furthermore, mechanistic study showed that Embinin activated the cAMP pathway in the rat models. In conclusion, Embinin has the potential to serve as a drug for the treatment of LDH.

## Graphical abstract

**Figure:**
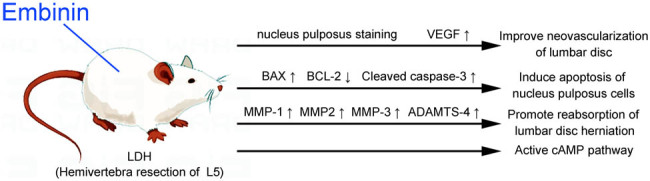

## 1 Introduction

The intervertebral discs lie between the vertebral bodies, linking them together. The components of the disc are nucleus pulposus (NP), annulus fibrosus, and cartilagenous end-plates [1]. The NP, encased by the fibrous annulus fibrosus and the cartilage end-plate, is an avascular structure. Reabsorption of the NP in lumbar disc herniation (LDH) refers to the natural absorption or even complete disappearance of LDH after conservative treatment. The reabsorption of LDH was first confirmed by computed tomography. The LDH reabsorption theory has brought new enlightenment for the prevention and treatment of LDH [2]. There are many mechanisms of LDH reabsorption, such as auto-immunity, inflammation, neovascularization, matrix metabolism imbalance, intervertebral disc degeneration, and NP cell apoptosis [3]. More studies have confirmed that signaling pathway-mediated apoptosis of NP cells may be an important mechanism of reabsorption [3]. NP cells will express a variety of inflammatory factors and stroma-degrading enzymes after contacting with blood supply, which will increase the apoptosis of NP cells, thus promoting the reabsorption of NP tissues [4]. In order to better treat LDH, further studies on its mechanism and developing new therapeutic drugs are crucial for the treatment of LDH.

Traditional Chinese medicine is critical in the treatment of LDH [5]. The study showed that moxibustion promoted absorption and motor function recovery of LDH in rats through the Fas/FasL pathway [6]. Demethoxycurcumin extracted from turmeric reduces the production of inflammatory factors in NP cells. Clematidis Radix Et Rhizoma is derived from the roots and rhizomes of *C. chinensis*, *C. hexapetala*, and *C. mandshurica*, which has the functions of removing wind dampness, clearing meridians, treating gout, obstinate arthralgia, and cold pain of waist and knee [7]. In orthopedic diseases, studies have found that Clematichinenoside (AR-6) from Wilingiaceae has antiarthritic effects on collagen-induced arthritis. Through TCMSP website analysis, Embinin is one of the ingredients of Wilingia (OB > 30, DL > 0.18). Kyoto encyclopedia of genes and genomes (KEGG) results showed that these targets are associated with neovascularization and apoptosis processes, which are closely related to the regulation of LDH reabsorption. Cyclic adenosine monophosphate (cAMP) is a second messenger that regulates a variety of signaling pathways. cAMP signaling is vital for neurite outgrowth and axonal guidance. Several studies have confirmed that cAMP signaling pathway is involved in the progression of LDH.

However, the possible effects of Embinin on the progression of LDH are still unclear. Therefore, the aim of this study was to clarify the role of Embinin in LDH rat models. The results showed that Embinin could affect the progression of LDH, and Embinin has the potential to serve as a therapeutic agent for LDH.

## 2 Materials and methods

### 2.1 LDH models

A total of 24 male SD rats (7 weeks old, 230–250 g) were divided into 4 groups with 6 rats in each group. In the model group, the LDH model was established by lumbar epidural insertion of the tail disc. The method is as follows: in short, 2–3% isoflurane gas anesthetized the rats and performed an L5 hemivertebrae excision on the right side with a fine sponge needle to expose the L5-6 discs. The L5-6 disc was punctured with a 30-G needle at a depth of 4 mm from the disc surface. Animals in sham received only hemivertebrae resection at the right L5 level. The spinal cord is then covered with a absorbable tourniquet (Surgicel fabric, Johnson and Johnson, Arlington, TX). All rats received an intramuscular injection of 40 mg/kg Cefazolin sodium after suturing. Animals also received 10 mg/kg oral acetaminophen syrup. Subsequently, rats in the administration group were intraperitoneally injected with different concentrations of Embinin (20 and 40 mg/ml, bought from Sigma, USA) twice a day for 10 days. All animals were sacrificed by 3% isoflurane gas and the lumbar spine containing the L5-6 disc was isolated.


**Ethical approval:** The research related to animal use has been complied with all the relevant national regulations and institutional policies for the care and use of animals, and has been approved by the Experimental Animal Ethics Committee of Wenzhou Medical University (Approval no. wydw. 2023-0147) and conducted in accordance with the National Institutes of Health Laboratory Animal Care and Use Guidelines.

### 2.2 Bioinformatic analysis

ChEMBL (https://www.ebi.ac.uk/chembl/CHEMBL) predicted drug targets, STRING (https://cn.string-db.org/) predicted drug target interactions, and R predicted GO and KEGG enrichment of drug targets, all of which were conducted on the corresponding websites.

### 2.3 Histological analysis

The lumbar spine tissues containing the L5-6 disc and DRG neurons were collected and then fixed with 4% paraformaldehyde at 4°C overnight. Spine samples were decalcified in decalcification solution until the spine is soft. Then, they were washed in PBS and cryoprotected with 30% sucrose for 3 days. Then, specimens were cut into 20-µm thicknesses, followed by embedded with paraffin and cut into sections. The sections were counterstained with hematoxylin-eosin (H&E) [7].

Immunohistochemistry was used to stain the puncture site of the L5-6 disc. Primary antibody against CD34 (ab81289, 1:500, Abcam, Cambridge, UK) was incubated with the sections at 4°C overnight. After washing three times with PBS, secondary antibodies were added (diluted to 1:300). After 2 h, the sections were washed three times with PBS. The stained tissue sections were photographed with confocal microscopy. The number of CD34-positive cells was quantified manually.

### 2.4 Flow cytometry (FCM) assays

NP tissue homogenate was determined with Annexin V and PI double staining kit (Beyotime, Beijing, China). The analysis was performed with a flow cytometer (FACSCalibur, BD, Franklin Lake, New Jersey, USA).

### 2.5 ELISA

The serum levels of VEGF and cAMP were assessed by ELISA kit (Abcam, UK). Briefly, samples were aspirated into wells. And biotin-conjugated primary antibodies were added into wells before the addition of avidin-conjugated HRP. Then, enzyme substrate was added for color development, and the result was measured with a microplate reader.

### 2.6 Immunoblot assay

The proteins from L4–L5 DRG were separated by a 10% SDS-PAGE experiment, and then, the total proteins were transferred onto PVDF membranes (Millipore, USA). Then, the PVDF membranes were blocked by the use of 5% dry milk and antibodies of Bax (1:1,000, ab32503; Abcam, UK, apoptosis), Bcl-2 (1:1,000, ab182858; Abcam, UK, apoptosis), Cleaved caspase-3 (1:1,000, ab32402; Abcam, UK, apoptosis), MMP-1 (1:1,000, ab52631; Abcam, UK, Cleaves collagens), MMP-2 (1:1,000, ab92536; Abcam, UK, Cleaves collagens), MMP-3 (1:1,000, ab52915; Abcam, UK, Cleaves collagens), ADAMTS4 (1:1,000, ab185722; Abcam, UK, Cleaves aggrecan), PKA (1:1,000, ab187515; Abcam, UK, cAMP signaling), CREB (1:1,000, ab32515; Abcam, UK, cAMP signaling), p-CREB (1:1,000, ab32096; Abcam, UK), and β-actin (1:1,000, ab8226; Abcam, UK) at 4°C overnight. The membranes were treated with the secondary antibodies for 45 min. Each blot was then visualized using the ECL kit (GE, SA).

### 2.7 Statistical analysis

Data were represented by mean ± SD. Statistical analysis was performed using GraphPad. *p* < 0.05 was considered significance. One-way ANOVA and Tukey’s post hoc test were used in this study.

## 3 Results

### 3.1 Embinin promotes lumbar disc neovascularization in LDH rats

**Figure 1:**
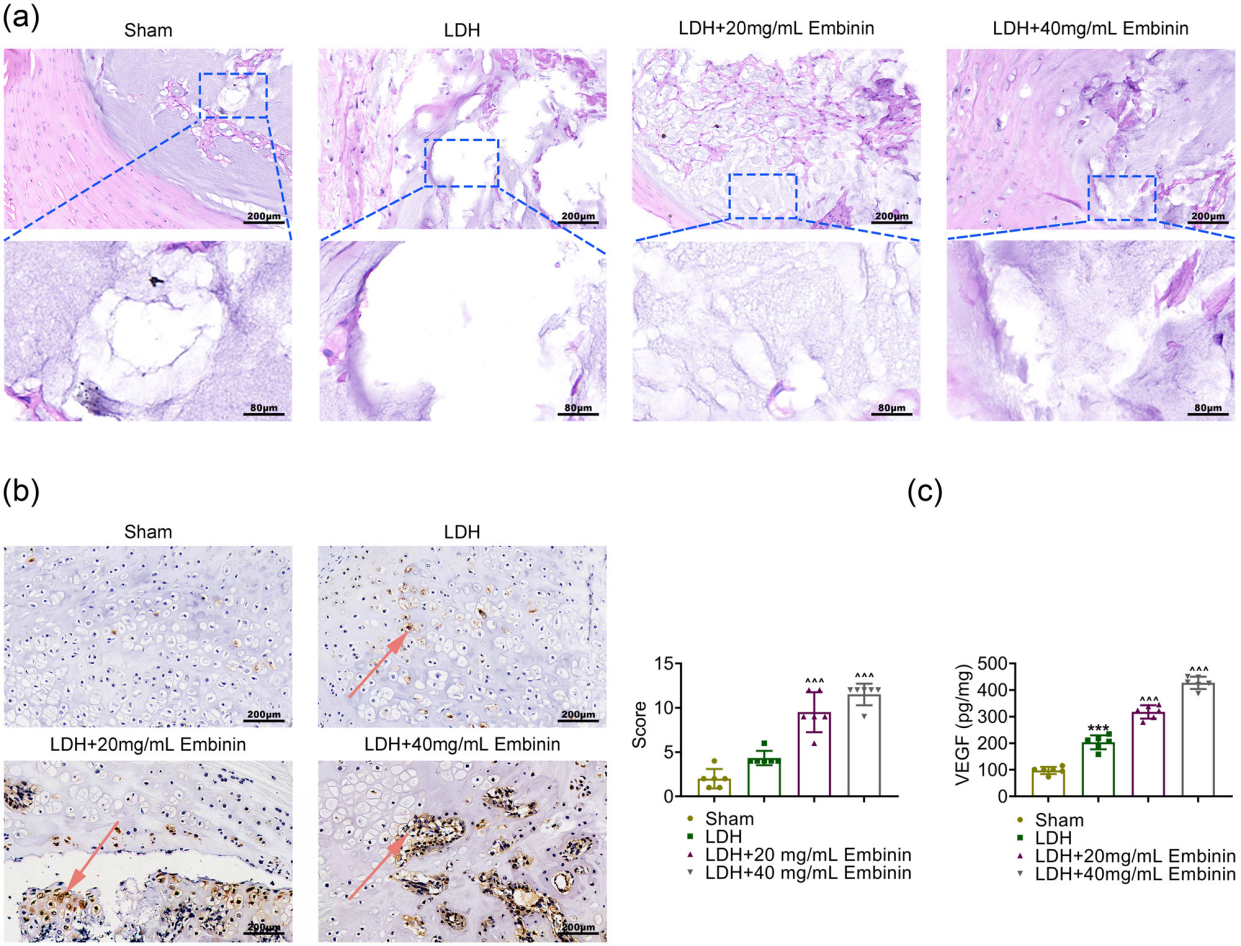
Embinin promotes lumbar disc neovascularization in LDH rats. (a) H&E staining showed the nucleus pulposus tissues of rats upon the indicated treatment. Significant morphological changes and degeneration of the nucleus pulposus were observed in the LDH group, and the amount of NP within the disc was dramatically decreased after surgery. Scale bar, 200 μm. (b) IHC assays showed the expression of CD34 in the nucleus pulposus tissues of rats upon the indicated treatment. The percentage of CD34-positive cells were calculated. Scale bar, 200 μm. The arrows indicate the positive signal. (c) ELISA assays showed the secretion of VEGF in the nucleus pulposus tissues of rats upon the indicated treatment. Error bars indicate SD. ***p < 0.001, LDH vs sham, ^^*p* < 0.01, ^^^*p* < 0.001, LDH + Embinin vs LDH.

LDH model with lumbar epidural insertion of the tail disc was first established. Subsequently, rats in the treatment group were intraperitoneally injected with different concentrations of Embinin (20 and 40 mg/ml). Through H&E staining, significant morphological changes and degeneration of the NP were observed in the LDH group, and the amount of NP within the disc was dramatically decreased after surgery (Figure 1a). However, morphological changes and degeneration of the NP were attenuated in Embinin groups (Figure 1a). Further through IHC assays, CD34-positive cells in the NP were increased in LDH rats (Figure 1b). Embinin treatment significantly increased the number of CD34-positive cells in LDH rats, suggesting the promotion of neovascularization (Figure 1b). Further through ELISA assays, LDH rats had more VEGF secretion than normal rats in the NP, and Embinin treatment significantly promoted the secretion of VEGF in LDH rats (Figure 1c). Therefore, Embinin promoted lumbar disc neovascularization in LDH rats.

### 3.2 Embinin-induced apoptosis of NP cells in LDH rats

**Figure 2:**
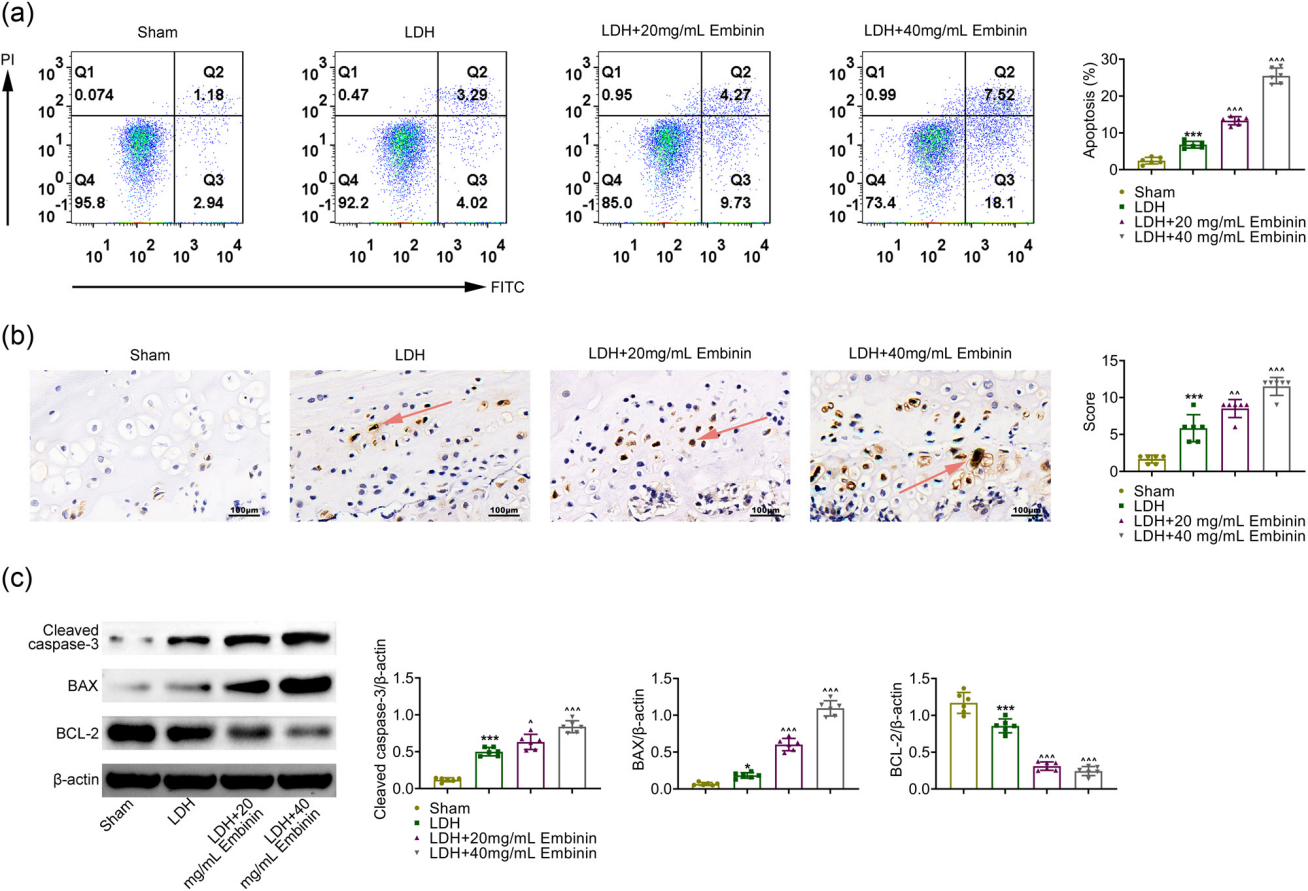
Embinin-induced apoptosis of nucleus pulposus cells in LDH rats. (a) FCM assays showed the apoptosis of nucleus pulposus tissue homogenate in rats upon the indicated treatment. (b) TUNEL assays showed the apoptosis levels of the nucleus pulposus tissues of rats upon the indicated treatment. Scale bar, 100 μm. The arrows indicate the positive signal. (c) Immunoblot assays showed the expression of cleaved caspase-3, Bax, and Bcl-2 in the nucleus pulposus tissues of rats upon the indicated treatment. Error bars indicate SD. *p < 0.05, ***p < 0.001, LDH vs sham, ^*p* < 0.05, ^^^*p* < 0.001, LDH + Embinin vs LDH.

We then detected whether Embinin could affect the apoptosis of NP cells. FCM assays indicated the induction of apoptosis of NP tissue homogenate in LDH rats, and Embinin further induced cell apoptosis in LDH rats (Figure 2a). Through TUNEL assays, the induction of apoptosis in LDH rats was noticed (Figure 2b). Embinin further induced cell apoptosis in LDH rats (Figure 2b). Immunoblot assays showed that the expressions of Bax and cleaved caspase-3 were increased in the NP cells from LDH rats, while Bcl-2 expression was decreased (Figure 2c). However, Embinin treatment further promoted the expression of Bax and cleaved caspase-3 and suppressed the expression of Bcl-2 in the NP cells of LDH rats (Figure 2c). Collectively, Embinin induced apoptosis of NP cells in LDH rats.

### 3.3 Embinin promotes lumbar disc resorption in LDH rats

**Figure 3:**
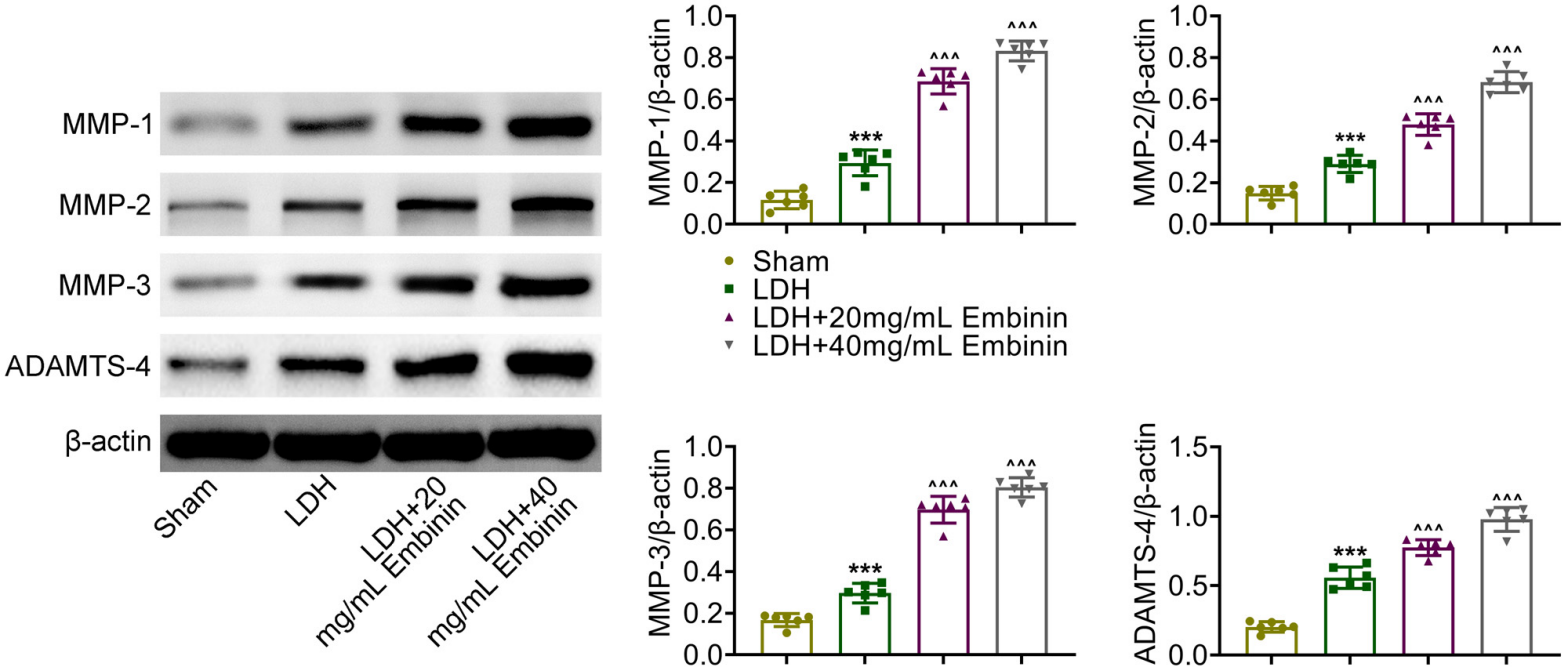
Embinin promotes lumbar disc resorption in LDH rats. Immunoblot assays showed the expression of MMP-1, MMP-2, MMP-3, as well as ADAMTS4 in the nucleus pulposus tissues of rats upon the indicated treatment. Error bars indicate SD. ***p < 0.001, LDH vs sham, ^^^*p* < 0.001, LDH + Embinin vs LDH.

Subsequently, whether Embinin promotes lumbar disc resorption in LDH rats was detected. MMP-1, MMP-2, MMP-3, and ADAMTS4 were the main proteins of extracellular matrix (ECM) and also markers of lumbar disc resorption. Through immunoblot assays, the expressions of MMP-1, MMP-2, MMP-3, and ADAMTS4 were all increased in the NP of LDH rats (Figure 3). Embinin treatment further increased the expression of these proteins in the NP from LDH rats (Figure 3). Therefore, Embinin promotes lumbar disc resorption in LDH rats.

### 3.4 Embinin activates the cAMP pathway in LDH rats

**Figure 4:**
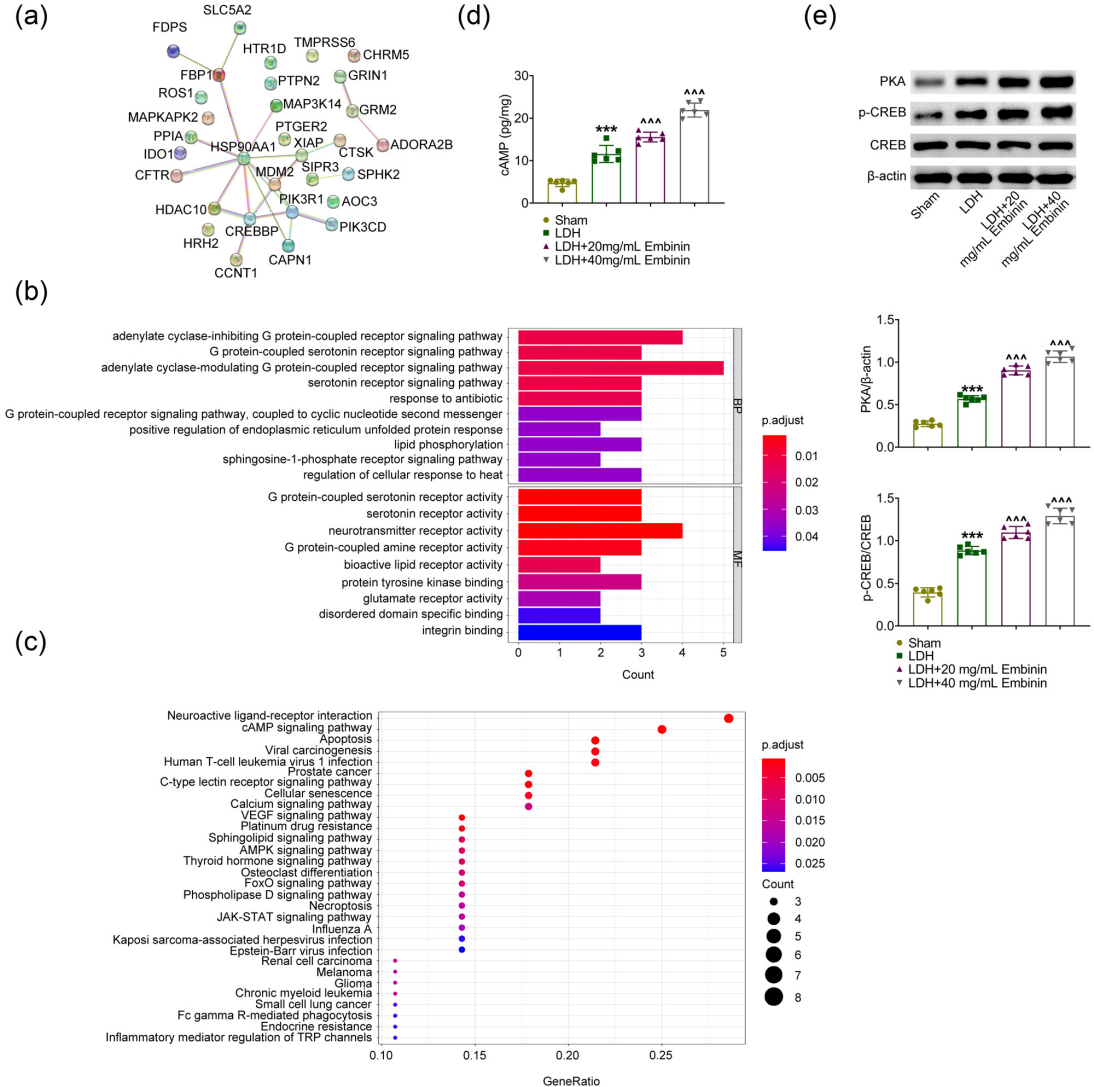
Embinin activates the cAMP signaling pathway in LDH rats. (a) PPI network diagram of 31 targets of Embinin. (b) GO enrichment analysis showed that the targets were enriched in G protein-coupled receptor signaling pathways. (c) KEGG analysis revealed enriched signaling pathways. (d) ELISA assays showed the secretion of cAMP in nucleus pulposus tissues of rats upon the indicated treatment. (e) Immunoblot assays showed the expression of PKA and CREB and phosphorylation levels of CREB in nucleus pulposus tissues of rats upon the indicated treatment. Error bars indicate SD. ***p < 0.001, LDH vs sham, ^^^*p* < 0.001, LDH + Embinin vs LDH.

The mechanism underlying Embinin suppressing the progression of LDH was explored. Embinin targets were predicted by ChEMBL and drug target interactions were predicted by STRING. Embinin has a total of 31 active targets, and the PPI network diagram of 31 targets are shown in Figure 4a. The GO analysis showed that these targets regulated by Embinin are enriched in G protein-coupled receptor signaling pathways (Figure 4b). Similarly, KEGG pathway analysis showed that these Embinin-related targets were enriched in the cAMP pathway, apoptosis, and neovascularization pathways, further confirming our previous results (Figure 4c). Then, the effects of Embinin on the cAMP pathway were detected. Through ELISA assays, Embinin obviously increased the secretion of cAMP in the NP from LDH rats (Figure 4d). In addition, the expression of PKA and the phosphorylation of CREB in the cAMP pathway were increased in LDH rats compared to sham, and the expression of PKA and the phosphorylation of CREB were further increased upon Embinin treatment in LDH rats (Figure 4e). Therefore, Embinin activates the cAMP pathway in LDH rats.

## 4 Discussion

LDH is a syndrome caused by disc degeneration, annulus fibrosus rupture, NP protrusion stimulation or compression of nerve roots, and cauda equina nerve, and it is one of the most common causes of back and leg pain [8]. The treatment of LDH can be divided into conservative treatment and surgical treatment [9]. New drugs are still needed to be explored. NP is a non-vascular cartilaginous tissue containing proteoglycan-rich ECM protein. AF is a ligamentous laminar structure wrapped around NP [9]. Lumbar disc degeneration is characterized by decreased water content and ECM breakdown, and NP cells that produce chondro-specific ECM components play an increasingly important role in the degeneration [10,11]. Abnormal apoptosis of NP cells is considered to be the main cellular process related to LDH [10,12,13]. Embinin induced apoptosis of NP cells and promoted LDH reabsorption in LDH rats, therefore suppressing the progression of LDH. The cellular composition of NP differs among species. Adult humans have chondrocyte-like cells, whereas adult rats have notochordal cells [14].

In addition to inflammation, neovascularization may be a key determinant of disc resorption because neovascularization usually occurs around a herniated disc [15]. In addition, VEGF, an important mediator of neovascularization, appears to be present in disc herniation tissue and can be released after disc herniation, thus promoting necessary neovascularization [16,17,18]. VEGF forms a vaso-neural network in and around the degenerative LDH, which provides material pathways for the resorption response [16]. Vascularization plays an important role in the resorption of herniated intervertebral disc tissue [16]. Previous studies have shown that the higher the degree of vascularization of herniated disc tissue, the more obvious the spontaneous absorption [19]. VEGF and CD34 have been shown to be associated with neovascularization in herniated disc tissue [19]. Our results confirmed that Embinin promoted lumbar disc neovascularization, but further *in vitro* and *in vivo* assays were needed to confirm our results.

In this study, a rat model of LDH was established by lumbar epidural insertion of the tail disc. Through IHC and Immunoblot assays, Embinin promoted lumbar disc neovascularization. Further through TUNEL and Immunoblot assays, Embinin induced apoptosis of NP cells and promoted LDH reabsorption in LDH rats. Through bioinformatic analysis, Embinin activated the cAMP signaling pathway in the LDH model. Therefore, LDH has the potential to serve as a drug for the treatment of LDH [20]. A previous study indicated that thermal and wine processing enhanced the efficacy of Clematidis Radix et Rhizoma to improve rheumatoid arthritis in rats [20]. Anti-arthritic effects of clematichinenoside (AR-6) on the PI3K/Akt pathway and TNF-α associated with collagen-induced arthritis were also found in a previous study [7]. To predict the targets of Embinin, KEGG results showed that these targets are associated with neovascularization and apoptosis processes, which are closely related to the regulation of LDH reabsorption. Our data further confirmed the effects of Embinin on the neovascularization and the apoptosis of NP cells. Several studies have confirmed that the cAMP signaling pathway is involved in the progression of LDH, and similarly, Embinin affects LDH through this signaling pathway [21]. However, the precise mechanism needs further study. Given that this is a new drug with few studies, further studies are needed to clarify the physiological effects of Embinin in different diseases, and in-depth research and discussion on its mechanism are also needed.

In summary, Embinin can promote the neovascularization and the apoptosis of NP cells in the lumbar intervertebral disc, thereby reabsorbing lumbar intervertebral disc herniation and relieving LDH symptoms. A mechanistic study demonstrated that its regulatory mechanism is related to the activation of the cAMP signaling pathway. Therefore, Embinin has the potential to serve as a drug for the treatment of LDH.
